# Relationship between Genotypes *Sult1a2* and *Cyp2d6* and Tamoxifen Metabolism in Breast Cancer Patients

**DOI:** 10.1371/journal.pone.0070183

**Published:** 2013-07-29

**Authors:** Ana Fernández-Santander, María Gaibar, Apolonia Novillo, Alicia Romero-Lorca, Margarita Rubio, Luis Miguel Chicharro, Armando Tejerina, Fernando Bandrés

**Affiliations:** 1 Department of Basic Biomedical Sciences, Faculty of Biomedical Sciences, Cátedra Florencio Tejerina-Universidad Europea de Madrid, Universidad Europea de Madrid, Madrid, Spain; 2 Department of Applied Medical Specialities, Psychology and Education, Faculty of Biomedical Sciences, Universidad Europea de Madrid, Madrid, Spain; 3 Department of Morphological Sciences, Faculty of Health Sciences, Universidad Europea de Madrid, Madrid, Spain; 4 School of Advanced Studies, Cátedra Florencio Tejerina-Universidad Europea de Madrid, Fundación Tejerina, Madrid, Spain; IPO, Inst Port Oncology, Portugal

## Abstract

Tamoxifen is a pro-drug widely used in breast cancer patients to prevent tumor recurrence. Prior work has revealed a role of cytochrome and sulfotransferase enzymes in tamoxifen metabolism. In this descriptive study, correlations were examined between concentrations of tamoxifen metabolites and genotypes for *CYP2D6, CYP3A4, CYP3A5, SULT1A1, SULT1A2* and *SULT1E1* in 135 patients with estrogen receptor-positive breast cancer. Patients were genotyped using the Roche-AmpliChip® CYP450 Test, and Real-Time and conventional PCR-RFLP. Plasma tamoxifen, 4-hydroxy-tamoxifen, N-desmethyl-tamoxifen, endoxifen and tamoxifen-N-oxide were isolated and quantified using a high-pressure liquid chromatography-tandem mass spectrometry system. Significantly higher endoxifen levels were detected in patients with the wt/wt *CYP2D6* compared to the v/v *CYP2D6* genotype (p<0.001). No differences were detected in the remaining tamoxifen metabolites among *CYP2D6* genotypes. Patients featuring the SULT1A2*2 and SULT1A2*3 alleles showed significantly higher plasma levels of 4-hydroxy-tamoxifen and endoxifen (p = 0.025 and p = 0.006, respectively), as likely substrates of the SULT1A2 enzyme. Our observations indicate that besides the *CYP2D6* genotype leading to tamoxifen conversion to potent hydroxylated metabolites in a manner consistent with a gene-dose effect, *SULT1A2* also seems to play a role in maintaining optimal levels of both 4-hydroxy-tamoxifen and endoxifen.

## Introduction

Tamoxifen (TAM) is widely used to prevent recurrence in patients with estrogen or progesterone receptor-positive breast cancer (BC) due to its estrogen receptor blocking effect [1]. Tamoxifen is described as a pro-drug since two of its metabolites, 4-hydroxy-tamoxifen (4OH-TAM) and *N*-desmethyl-4-hydroxy-tamoxifen (endoxifen), both have an affinity for the estrogen receptor that markedly exceeds that of TAM itself [2]. Endoxifen is considered the main active metabolite of TAM, since its estrogen receptor affinity is 100-fold that of TAM and its serum levels are 10-fold those of 4OH-TAM [3].

Tamoxifen is metabolized via the cytochrome P450-mediated pathway to several primary and secondary metabolites that show variable potency toward the estrogen receptor. *N*-desmethyltamoxifen (NDM-TAM), produced by CYP3A4/5-mediated metabolism, is the major primary metabolite, accounting for 90% of primary TAM oxidation, whereas 4OH-TAM, mediated by CYP2D6 activity, is a minor metabolite. Both NDM-TAM (produced via CYP2D6) and 4OH-TAM (produced via CYP3A4/5) are secondarily metabolized to endoxifen [4]. Although it has been established that tamoxifen and its metabolites undergo phase II conjugation reactions including glucuronidation and sulfation, few studies have examined the role of phase II sulfotransferase enzymes (SULTs) in TAM metabolism. Given that 4-OH-TAM and endoxifen are known substrates of SULT1A1 [5 and 6, respectively], different SULT enzyme activity levels could markedly influence the efficacy of TAM [5].

CYP-regulated drug metabolism is prone to genetic variability, which can lead to normal, low or null activity levels of a given enzyme. The main enzyme responsible for 4OH-TAM and endoxifen formation, CYP2D6 [7], is highly polymorphic. Over 80 different alleles resulting in reduced or impaired CYP2D6 activity have been reported [8]. Subjects carrying two null CYP2D6 alleles are classed as poor drug metabolizers (PMs), with 5–10% of Caucasians classified as PMs [9]. The CYP2D6*4 allele, followed by CYP2D6*5, CYP2D6*3 and CYP2D6*6, is the main null allele that gives rise to the PM phenotype in Europeans (12–22%) [10]. Around eighty single nucleotide polymorphisms (SNPs) of *CYP3A4/5* have been reported to the Human P450 Allele Nomenclature Committee [8]. The CYP3A5*3 SNP at intron 3 causes alternative protein splicing and truncation [11]. This mutant allele is considered the main defective *CYP3A5* allele and frequencies of this allele as high as 95% have been described in some European populations [12], [13]. This variant plays an important role in interindividual and interethnic differences in the metabolic profiles of many drugs [14]. Two inactive genetic variants of *CYP3A4*, CYP3A4*3 and CYP3A4*17, have also been described in Caucasian populations [15], [16]. The CYP3A4*3 allele, which has a T1473C change that produces a Met445Thr substitution in exon 12, induces structural differences in the enzyme modifying its activity. The CYP3A4*17 polymorphism with a T>C mutation in exon 7 is a putative defective allele that leads to >99% reduction in catalytic activity [15]. Breast cancer patients under treatment with TAM who feature the CYP3A4*1B allele and a single A to G change in the promoter may be at an increased risk of developing endometrial cancer, as described by Chu et al. [17]. Recently, a lower production of NDM-TAM in CYP3A5*3/*3 microsomes compared to the wild-type genotype has been described by Mugundu et al. [18]. However, so far no variant *CYP3A4* allele has been linked to a modified TAM metabolism. Some authors have reported considerable interindividual variation in plasma levels of TAM metabolites that could affect the response to treatment [19, for instance]. Hence, genetic variability in the genes coding for the enzymes CYP2D6, CYP3A4 and CYP3A5 could explain such variations in metabolite concentrations.

Sulfotransferase enzymes are a family of phase II liver enzymes involved in the detoxification of a variety of xenobiotic and endogenous compounds. These enzymes catalyze the transfer of a sulfonyl group to nucleophilic groups increasing their solubility and facilitating their excretion. SULT1A1 is the most highly expressed SULT in the liver and some studies have shown that the high-activity SULT1A1*1 allele is linked to a better overall survival rate in BC patients receiving TAM [7]. Among all known SULTs, SULT1E1 shows the highest affinity for estrogens indicating its activity at physiologically significant estrogen concentrations [20]. Moreover, *SULT1E1* is highly expressed in normal human mammary epithelial cells [21] and may play an important role in estrogen-driven BC development. In effect, genetic polymorphisms in *SULT1E1* have been associated with both an increased risk of BC and disease-free survival in Asian women [22]. Further, a study examining the role of the sulfotransferase gene, *SULT1A2*, has revealed its contribution to the TAM-resistant phenotype in the presence of certain combinations of *CYP2C9* and *SULT1A2* allelic variants [23].

Given the complexity of TAM metabolism and the inconsistent results provided in the literature, this descriptive study was designed to examine relationships between TAM metabolite concentrations and genotypes for *CYP2D6*, *CYP3A4*, *CYP3A5*, *SULT1A1*, *SULT1A2* and *SULT1E1* in 135 patients with estrogen receptor-positive breast cancer. Besides our findings related to the *CYP2D6* genotype, this paper presents the first data on the effects of *SULT1A2* genotypes on TAM metabolite levels.

## Patients and Methods

### Ethics Statement

The study protocol was approved by the Review Board of the Hospital de Getafe (Madrid, Spain). Written informed consent to participate in the study was obtained from all participants.

### Patients

One hundred and thirty five Caucasian patients of Spanish descent recently diagnosed with BC were recruited from the center *Fundación Tejerina-Centro de Patología de la Mama* (Madrid). Premenopausal and postmenopausal women were enrolled when started on TAM as standard adjuvant therapy after undergoing primary surgery, radiation and adjuvant chemotherapy. Patients were excluded if they had started TAM therapy simultaneously with either adjuvant chemotherapy or adjuvant radiation therapy (or both) or if they were undergoing other adjuvant endocrine therapies. Patients who were pregnant or breast-feeding were also excluded from the study. Enrolled patients were allowed to take vitamin E, selective serotonin reuptake inhibitors (SSRIs) or herbal remedies. Blood samples were collected within 3 to 60 months of initiating TAM treatment.

### Samples

Venous blood was collected from all 135 subjects before taking their daily 20- mg dose of TAM. Ten milliliters of heparin plasma were separated by centrifugation and immediately stored at −20°C until analysis. In addition, one milliliter of an EDTA blood sample was taken for subsequent DNA extraction and genotyping. DNA was isolated from peripheral leukocytes using a QIAamp DNA Blood Mini Kit® (Qiagen, Madrid, Spain) according to the manufacturer’s instructions. The DNA concentration was determined and adjusted to 2–20 ng/ µL.

### Genotyping

The Roche-AmpliChip® CYP450 Test (Roche, Spain) was used to identify 33 *CYP2D6* alleles (including duplications and deletions) in the plasma samples. The AmpliChip CYP450 Test microarray serves to examine both sense and antisense strands of an amplified target DNA sample. The test was powered by Affymetrix microarray technology. In most patients, the presence of common mutations was confirmed using a different genotyping method: a long PCR product was used as a template to type alleles in separate multiplex allele-specific PCRs, based on SBE with fluorescent labeled ddNTPs (ABI Prism SNaPshot Multiplex Kit, Applied Biosystems, USA).

The CYP3A4*3 variant allele was determined by PCR, using the forward primer 5′-TGG ACC CAG AAA CTG CAT ATG C-3 and reverse primer 5′- GAT CAC AGA TGG GCC TAA TTG-3 under the PCR conditions described by van Schack et al. [16]. The nucleotides underlined are mismatches with the normal *CYP3A4* sequence that create a *Nsi*I restriction site in the wild-type *CYP3A4* PCR product. The CYP3A4*17 variant allele was also identified by PCR, using the forward primer 5′-CTGGACATGTGGGTTTCCTGT-3′ and reverse primer 5′- AGCAGTTATTTTTAAGAGAGAAAGATAAAT-3′ followed by digestion with the *Bpm*I restriction enzyme as described by Lee et al. [15]. The CYP3A4*1B polymorphism, a single A to G transition in the *CYP3A4* promoter, was detected using the ABI Prism 7700 Sequence Detection System and the standard SDS allele discrimination protocol. To this end, a 104- bp PCR product was amplified using the forward and reverse primers 5′-GTGTGGCTTGTTGGGATGAA-3 and ´5′-GTGGAGCCATTGGCATAAAAT-3, respectively, and detected using the fluorescently-labeled probes 5′-6-carboxy-fluorescein (FAM)-AATCGCCTCTCTCtTGCCCTTGTCTCT-BHQ1–3 and HEX-AATCG CCTCTCTCcTGCCCTTGTCT-BHQ1–3 for the A and G alleles, respectively, according to the method of Spurdle et al. [24]. Results were confirmed by RFLP using the forward 5′-GGACAGCCATAGAGACAAGGGGA- 3′ and reverse 5-CACTCACTGACCTCCTTTGAGTTCA-3′ primers followed by digestion with the *Mbo*II enzyme [25]. CYP3A5*3 was detected by the PCR-RFLP procedure described by van Schaik et al. [13] using the *Ssp*l restriction enzyme and the forward and reverse primers 5′-CATCAGTTAGTAGACAGATGA-3′ and 5′-GGTCCAAACAGGGAAGAAATA-3′, respectively.

For the *SULT1A1* gene, alleles *1 and *2 were identified by PCR-RFLP using the *Hae*II enzyme and the forward and reverse primers 5′-GGTTGAGGAGTTGGCTCTGC-3′ and 5′-ATGAACTCCTGGGGGACGGT-3′ respectively under the PCR conditions described in Coughtrie et al. [26]. For the *SULT1A2* gene, two pairs of primers and two different restriction enzymes were used to genotype alleles *1 (wt), *2 and *3, as described by Arslan et al. [27]. The PCR product amplified with the primers F5′-GAACATGGAGCTGATCCAGGTC-3′ and R5′-CTGAGGTGAGCATGACCTCG-3′ was digested with the *BstE*II enzyme and the product amplified with F5′-GGAACCACCACATTAGAGC-3′ and R5′-GCCTCTGCAAAGTACTTGATGCG-3′ was digested with *BstU*I. To genotype the *SULT1E1* gene, samples were amplified with the primers F 5′-CTCCTTCTCTGGCATTCAGG-3′ and R 5′-CAACCTGTTTAGTTGATCCTGTG-3′ and digested with the *Dde*I enzyme, as described by Adjei et al. [28].

Any mutations detected were confirmed by repeating the procedure or using a different technique whenever possible. Samples were discarded if there was disagreement between the methods or repetitions.

### Quantifying Tamoxifen and its Metabolites in Plasma

#### Reagents and chemicals

Tamoxifen, 4-hydroxy-tamoxifen, N-desmethyl-tamoxifen, N-desmethyl-4-hydroxy-tamoxifen (1∶1 E/Z mixture), tamoxifen-N-oxide, tamoxifen-d5, 4-hydroxy-tamoxifen-d5, N-desmethyl-tamoxifen-d5, and N-desmethyl-4-hydroxy-tamoxifen-d5 (1∶1 E/Z mixture) were purchased from Toronto Research Chemicals (North York, Ontario, Canada).

Acetonitrile, methanol, distilled water and formic acid were obtained from Fluka Analytical (Sigma-Aldrich, Spain). All chemicals used were of analytical grade. Small (1 mL) volumes of drug-free human serum were pooled and used for validation purposes.

#### HPLC

TAM and its metabolites were separated and quantified by high-pressure liquid chromatography-tandem mass spectrometry using an Agilent HPLC 1200 system. HPLC experiments were performed using a binary pump G1312A, a G1316A column oven, G1379B degasser and an automatic injector H-ALS G1367B. Mobile phase A and phase B consisted of 0.1% formic acid in water and acetonitrile, respectively. Mobile phases A and B were pumped through a ZORBAX Eclipse XDB-C18 column (150 mm×2.1 mm I.D., 3.5 µm, Agilent USA) at a flow rate of 0.2 mL/min using the gradient shown in Table 1. Separation was conducted at 30°C. 15- µL aliquots were injected and the autosampler needle was rinsed in acetonitrile/water solution (1∶1). The total run time was 20 min. During the first 4.0 and last 2.0 min, the eluate was removed using a divert valve to avoid endogenous compounds entering the mass spectrometer.

**Table 1:** HPLC gradient parameters used to separate tamoxifen and its metabolites using a ZORBAX Eclipse XDB-C18 column (150 mm x 2.1 mm I.D., 3.5 µm) at 30°C.

Time (minutes)	Flow rate (mL/min)	Mobile phase A^a^ (%)	Mobile phase B^b^ (%)
0	0.2	65	35
7	0.2	50	50
15	0.2	25	75
20	0.2	65	35

a Mobile phase A: 0.1% formic acid in water.

b Mobile phase B: acetonitrile.

As a detector, we used an A 6410 Triple Quadrupole mass spectrometer (Agilent Technologies, USA) equipped with a heated electrospray ionization source (Thermo Fisher Scientific, Waltham, MA, USA) operating in positive ion mode. For quantification, multiple reaction monitoring (MRM) chromatograms were acquired using Mass Hunter software version B.01.04 (Agilent Technologies, USA). Positive ions were created at atmospheric pressure. Quadrupoles operated at unit resolution (0.7 Da). The HESI/MS/MS operating parameters and mass transitions are provided in Table 2.

**Table 2:** HPLC and MS parameters used to discriminate tamoxifen and its metabolites.

Compound	Precursorion (amu)	Production (amu)	Dwell(ms)	Fragmentor(v)	Collision energy (v)	Retentiontime (min)	MRM^a^	LLOQ^b^ (nM)
Tamoxifen	372	72	50	160	25	12.57	372 → 72	0.38
4-Hydroxy-tamoxifen	388	72	50	160	25	8.75	388 → 72	0.35
N-desmethyl-tamoxifen	358	58	50	120	20	12.16	358 → 58	0.35
N-desmethyl-4-hydroxy-tamoxifen	374	58	50	140	20	8.33	374 → 58	0.48
Tamoxifen-N-oxide	388	72	50	160	25	13.14	388 → 72	0.35

a Multiple reaction monitoring.

b Lower limit of quantification.

### Calibration Standards, Quality Controls and Internal Standard Solutions

Two separate stock solutions of all analytes (1 mg/mL) and internal standards (1 mg/mL) were prepared by dissolving accurately-weighed approximate 1 mg amounts in 1 mL of methanol. One stock solution was used to prepare calibration standards and the other stock solution to prepare quality control standards. A mixture of internal standard stock solutions was prepared and this mixture was diluted in acetonitrile to obtain a working solution for sample pretreatment. This internal standard working solution contained: tamoxifen-d5, 4-hydroxy-tamoxifen-d5, N-desmethyl-tamoxifen-d5 and N-desmethyl-4-hydroxy-tamoxifen-d5 (1∶1 E/Z mixture).

### Sample preparation

A 300- µL volume of 1% formic acid in acetonitrile containing internal standards was added to a 100- µL serum aliquot. After vortexing and centrifugation, the clear supernatant was transferred to a HybridSPE column (Supelco) and the eluents stored at 2–8°C until analysis. Samples were analyzed in triplicate.

### Validation Procedures

Eleven non-zero calibration standards were prepared in duplicate for each run and analyzed in three independent runs. Calibration curves (area ratio obtained with the internal standard versus nominal concentration) were fitted by least-squares linear regression using the reciprocal of the squared concentration (1/×2) as a weighting factor. The intra- and inter-assay accuracy and precision of the method were determined by assaying three replicates of each of the quality control samples at the lower limit of quantification (LLQ), at a low, a medium and a high concentration level in three separate runs. The concentration of each quality control sample was calculated using the calibration standards that were analyzed in duplicate in the same run. Differences between nominal and measured concentrations were used to calculate accuracy. Accuracy should be within 85–115% and precision should not exceed 15% of the CV. Carry-over was determined by injecting a processed control human serum sample after an upper limit of quantification sample. Areas of peaks in the blank processed sample should be within 20% of the peak area of the LLQ sample. Four individual batches of control human serum were used to assess the specificity and selectivity of the method. To determine whether endogenous constituents interfere with the assay, a double blank and a sample spiked at the LLQ were processed from these batches.

### Data Analyses

Genotype frequencies, allele frequencies and Hardy-Weinberg equilibria were determined using the Genepop software package (v 4.1). Descriptive statistics were calculated using standard methods. Metabolic ratios were calculated as the concentration of substrate/concentration of metabolite. The non-normal distribution of data was confirmed by the Kolmogorov-Smirnov test. Levels of TAM, metabolites and ratios between genotype groups were compared using Wilcoxon-Mann-Whitney or Kruskal-Wallis tests. All statistical tests (two-sided) were performed using SPSS software (version 18.0 SPSS, Chicago, IL). Significance was set at a p<0.05.

## Results

### Demographics

The cohort examined was comprised of 135 Spanish patients with BC from different geographic regions. Mean age was 52.33 years (SD = 9.90, range 30 to 81 years). Tumor types were: 51.7% infiltrating ductal carcinoma, 29.9% ductal carcinoma in situ, 11.5% infiltrating lobular carcinoma and 6.9% both infiltrating ductal carcinoma and ductal carcinoma in situ. Seven patients (5.1%) were receiving concomitant CYP2D6 inhibitors (fluoxetine, paroxetine or citalopram) and five patients (3.6%) were receiving concomitant CYP2D6 substrates (propanolol and fluroxamine).

### Genotyping

*CYP2D6* genotype and allele frequencies are provided in Tables 3 and 4, respectively. The heterozygous polymorphism occurring at the highest frequency was wt/*4 (15.04%, Table 3). Null and intermediate allele frequencies of *CYP2D6* variants were as follows: 0.75% (*3), 11.65% (*4), 3.38% (*5), 0.38% (*6), 1.5% (*9), 0.38% (*10), 0.75% (*17), and 3.76% (*41) (Table 3). For the *CYP3A4* gene, low frequencies of the defective alleles CYP3A4*1B, CYP3A4*3 and CYP3A4*17 were observed (1.23%, 0.83% and 3.72%, respectively, Table 4). Conversely, a high incidence (97.78%) was detected of the null CYP3A5*3 allele (Table 4). Among the SULT genes, *SULT1A2* was the most polymorphic, showing up to 49.62% heterozygosity. Mutation frequencies in SULT genes were 30.08% (SULT1A1*2), 30.83% (SULT1A2*2), 15.79% (SULT1A2*3) and 15.04% (SULT1E1*2) (Table 4). All the CYP and SULT genes frequencies examined exhibited good agreement with Hardy-Weinberg equilibrium. The data for some patients (<3%) were discarded due to conflicting genotyping results.

**Table 3:** *CYP2D6*, *CYP3A4*, *CYP3A5*, *SULT1A1*, *SULT1A2* and *SULT1E1* genotype frequencies.

GENOTYPE (individuals)	FREQUENCY (%)
***CYP2D6*** * n = 133*	
wt/wt (+)	63.91
wt/*3	0.75
wt/*4	15.04
wt/*5	3.76
wt/*6	0.75
wt/*9	2.26
wt/*17	0.75
wt/*41	2.26
wtxN/*4	1.50
*3/*9	0.75
*4/*4	1.50
*4/*5	0.75
*4/*10	0.75
*4/*41	2.26
*5/*17	0.75
*5/*41	1.50
*41/*41	0.75
***CYP3A4*** * n = 121*	
wt/wt	88.40
wt/*1B	2.50
wt/*3	1.70
wt/*17	7.40
***CYP3A5*** * n = 135*	
wt/*3	4.44
*3/*3	95.56
***SULT1A1*** * n = 133*	
wt/wt	51.13
wt/*2	36.09
*2/*2	12.03
***SULT1A2*** * n = 133*	
wt/wt	33.83
wt/*2	25.56
*2/*2	12.78
wt/*3	13.53
*2/*3	10.53
*3/*3	3.76
***SULT1E1*** * n = 133*	
wt/wt	73.68
wt/*2	22.56
*2/*2	3.76

(+) “wt” allele correspond to normal enzyme activity. In the case of CYP2D6 wt includes *1, *2 and *35 alleles.

**Table 4:** *CYP2D6*, *CYP3A4*, *CYP3A5*, *SULT1A1*, *SULT1A2* and *SULT1E1* allele frequencies. International codes for SNPs between parentheses.

ALLELE	FREQUENCY (%)
***CYP2D6*** * n = 133*	
wt(+)	76.69
wtxN	0.75
*3 (rs35742686)	0.75
*4 (rs3892097)	11.65
*5	3.38
*6 (rs5030655)	0.38
*9 (rs5030656)	1.50
*10 (rs1065852)	0.38
*17 (rs28371706)	0.75
*41 (rs16947)	3.76
***CYP3A4*** * n = 121*	
wt	94.22
*1B (rs2740574)	1.23
*3 (rs4986910)	0.83
*17 (rs4987161)	3.72
***CYP3A5*** * n = 135*	
wt	2.22
*3 (rs776746)	97.78
***SULT1A1*** * n = 133*	
wt	69,90
*2 (rs6839)	30,08
***SULT1A2*** * n = 133*	
wt	53,38
*2 (rs1136703)	30,83
*3 (rs199986857)	15,79
***SULT1E1*** * n = 133*	
wt	84,96
*2 (rs3736599)	15,04

(+) “wt” allele correspond to normal enzyme activity. In the case of CYP2D6 wt includes *1, *2 and *35 alleles.

### Plasma Concentrations of Tamoxifen and its Metabolites

The data for some patients were eliminated because of conflicting results or technical problems. The mean TAM concentration of the samples analyzed (n = 125) was 202.32±94.55 ng/mL (median = 176.98 ng/mL). The mean NDM-TAM concentration was over 2 times the TAM concentration (450.54±188.79 ng/mL; median = 418.46 ng/mL). Of TAM’s known clinically active hydroxylated metabolites, endoxifen showed the highest mean concentration being almost three times the 4OH-TAM mean concentration (24.75±19.37 ng/mL and median = 19.11 ng/mL *vs*. 9.01±7.13 ng/mL and median = 6.75 ng/mL). The mean Tamoxifen-N-oxide level was 50.88±23.09 ng/mL (median = 48.81 ng/mL).

No significant differences were detected in mean plasma concentrations of TAM, NDM-TAM, 4OH-TAM and TAM-N-oxide between patients receiving CYP2D6 inhibitors concomitantly with TAM and those not receiving these inhibitors (180.10±61.25 ng/mL versus 203.63±96.19 ng/mL (p = 0.68)); 453.71±95.47 ng/mL versus 450.38±192.56 ng/mL (p = 0.60); 6.81±4.69 ng/mL versus 9.12±7.22 ng/mL (p = 0.46); 57.04±24.25 ng/mL versus 50.51±23.08 ng/mL (p = 0.44), respectively. However, mean plasma endoxifen concentrations were significantly lower in patients taking CYP2D6 inhibitors than those not taking these drugs (15.55±17.77 ng/mL versus 25.30±19.39 ng/mL (p = 0.03)). These findings reflect the importance of the CYP2D6 enzyme in the formation of endoxifen. However, no significant differences in mean plasma endoxifen concentrations were observed in patients under treatment with other CYP2D6 substrates (i.e., likely tamoxifen competitors) compared to those those not taking CYP2D6 substrates.

### Correlating CYP and SULT Genotypes with Plasma Metabolite Concentrations

In Table 5 we provide the means, standard deviations, medians and ranges of TAM and its metabolite concentrations for the *CYP2D6*, *CYP3A4* and *CYP3A5* genotypes. Due to *CYP2D6* gene variability, genotypes were classified according to pairs of alleles as different combinations of: “wt” including all extensive metabolizer alleles (CYP2D6*1, CYP2D6*2 and CYP2D6*35), “wtxN” including all ultraextensive metabolizer alleles (CYP2D6*1xN and CYP2D6*2xN), “P” including the null alleles (CYP2D6*3, CYP2D6*4, CYP2D6*5, CYP2D6*6, CYP2D6*7, CYP2D6*8) and “I” including intermediate metabolizer alleles (CYP2D6*9, CYP2D6*10, CYP2D6*17, CYP2D6*41). In this manner, 7 different *CYP2D6* genotypes were identified in the sample analyzed: wt/wt, wtxN/P, wt/P, wt/I, I/I, I/P and P/P (Table 5). Endoxifen was the only metabolite that varied significantly in the concentration among the different genotypes for *CYP2D6* (p = 0.026, Table 5). Product/substrate ratios were estimated for the two active hydroxylated TAM metabolites. Significant differences were observed among *CYP2D6* genotypes in plasma concentration ratios of endoxifen/NDM-TAM (p<0.001) but not of 4OH-TAM/TAM. No significant differences in TAM metabolite levels were observed among the genotypes for *CYP3A4* and *CYP3A5* (Table 5). For the *SULT1A1, SULT1A2* and *SULT1E1* genotypes, Table 6 provides the means, standard deviations, medians and ranges of TAM and its metabolite concentrations. This table indicates no significant differences in metabolite concentrations for the *SULT1A1* and *SULT1E1* genotypes. In contrast, endoxifen levels differed significantly among the *SULT1A2* genotypes (p = 0.027, Table 6) and 4OH-TAM levels showed a similar trend, albeit not significant (p = 0.056, Table 6), whereby higher concentrations of active metabolites (SULT1A2 substrates) were observed in patients carrying null alleles (*2, *3).

**Table 5:** Concentrations of tamoxifen and its metabolites (means (±SD), medians (in cursive) and ranges (in parentheses)) detected in patients with the *CYP2D6*, *CYP3A4* and *CYP3A5* genotypes.

Genotype	Tamoxifen (ng/mL)	4-OH-tamoxifen (ng/mL)	N-desmethyl-tamoxifen (ng/mL)	Endoxifen (ng/mL)	Tamoxifen-N-oxide (ng/mL)
**CYP2D6** (+)	203.05±80.53	9.49±7.44	423.00±179.76	26.47±17.89	50.38±23.51
**wt/wt**	*176.91*	*7.26*	*383.29*	*21.21*	*43.59*
n = 78	(77.10–573.83)	(1.06–35.90)	(35.96–1,191.13)	(4.03–95.87)	(15.69–142.97)
**CYP2D6** (+)	183.66±114.20	8.41±9.25	420.73±207.95	36.09±32.08	33.12±0.00
**wtxN/P**	*183.66*	*8.41*	*420.73*	*36.09*	*33.12*
n = 2	(102.91–264.41)	(1.87–14.95 )	(273.69–567.77)	(13.40–58.77)	(33.12–33.12)
**CYP2D6** (+)	209.57±121.53	8.91±7.71	501.59±201.33	23.76±25.21	54.04±25.63
**wt/P**	*169.64*	*6.05*	*461.18*	*14.57*	*55.79*
n = 26	(89.79–576.23)	(1.90–30.96)	(250.43–1,210.09)	(5.01–107.10)	(15.69–106.35)
**CYP2D6** (+)	208.48±66.06	6.77±4.50	502.47±213.05	26.87±17.31	55.21±12.62
**wt/I**	*189.98*	*5.61*	*482.28*	*20.64*	*48.81*
n = 4	(151.94–302.03)	(2.75–13.10)	(276.69–768.62 )	(14.01–52.20)	(47.07–69.74)
**CYP2D6** (+)	130.54±0.00	5.08±0.00	364.81±0.00	9.53±0.00	27.89±0.00
**I/I**	*130.54*	*5.08*	*364.81*	*9.53*	*27.89*
n = 1	(130.54–130.54)	(5.08–5.08)	(364.81–364.81)	(9.53–9.53)	(27.89–27.89)
**CYP2D6** (+)	173.84±65.10	6.56±1.25	488.85±194.12	12.01±5.84	43.32±20.74
**I/P**	*185.42*	*6.54*	*505.66*	*10.00*	*42.72*
n = 8	(88.08–289.96)	(4.87–8.19)	(271.13–847.98)	(4.46–21.02)	(20.53–76.71)
**CYP2D6** (+)	278.43±132.13	10.97±±0.00	746.52±313.81	8.71±6.41	60.15±30.82
**P/P**	*278.43*	*10.97*	*746.52*	*8.71*	*60.15*
n = 2	(185.00–371.83)	(10.97–10.97)	(524.62–968.42)	(4.18–13.24)	(38.85–81.94)
**CYP2D6 ** ***P value***	0.794	0.893	0.271	0.026	0.516
**CYP3A4**	198.93±89.59	9.09±6.90	440.17±180.39	24.80±19.03	50.14±22.26
**wt/wt**	*175.91*	*6.79*	*408.42*	*19.12*	*45.33*
n = 107	(77.10–576.23)	(1.06–30.96)	(35.96–1,210.09)	(4.03–107.10)	(15.69–111.58)
**CYP3A4**	260.69±136.19	5.88±3.02	641.07±223.51	22.31±10.86	62.77±12.19
wt/*1B	*261.00*	*7.20*	*756.86*	*26.69*	*68.00*
n = 3	(124.35–396.73)	(2.43–8.01)	(383.42–782.92)	(9.95–30.30)	(48.84–71.48)
**CYP3A4**	212.83±68.55	13.52±4.14	502.31±205.44	49.15±21.86	37.49±3.70
wt/*3	*212.83*	*13.52*	*502.31*	*49.15*	*37.49*
n = 2	(164.35–261.30)	(10.59–16.45)	(357.04–647.58)	(33.69–64.61)	(34.87–40.10)
**CYP3A4**	230.28±153.17	8.96±11.18	516.31±278.47	19.86±24.88	53.66±38.28
wt/*17	*187.36*	*5.00*	*429.19*	*11.94*	*34.87*
n = 9	(88.08–573.83)	(1.87–35.90)	(273.69–1,191.13)	(4.46–84.57)	(24.40–142.97)
**CYP3A4 ** ***P value***	0.776	0.308	0.320	0.086	0.400
**CYP3A5**	164.16±51.72	5.13±2.16	374.89±122.90	14.14±7.56	55.79±28.95
wt/*3	*160.17*	*5.32*	*345.54*	*13.61*	*47.07*
n = 6	(112.43–235.19)	(2.32–7.56)	(258.23–603.01)	(5.40–23.01)	(24.40–106.35)
**CYP3A5**	204.98±96.33	9.11±7.21	455.69±191.91	25.10±19.66	50.50±23.05
*3/*3	*176.98*	*6.83*	*425.25*	*19.12*	*47.07*
n = 117	(77.10–576.23)	(1.06–35.90)	(35.96–1,210.09)	(4.03–107.10)	(15.69–142.97)
**CYP3A5 ** ***P value***	0.385	0.224	0.261	0.142	0.672

(+) For the CYP2D6 genotypes:

“wt” includes all extensive metabolizer alleles (CYP2D6*1, CYP2D6*2, CYP2D6*35).

“wtxN” includes all ultraextensive metabolizer alleles (CYP2D6*1xN, CYP2D6*2xN).

“P” includes all null alleles (CYP2D6*3, CYP2D6*4, CYP2D6*5, CYP2D6*6, CYP2D6*7, CYP2D6*8).

“I” includes all intermediate metabolizer alleles (CYP2D6*9, CYP2D6*10, CYP2D6*17, CYP2D6*41).

**Table 6:** Concentrations of tamoxifen and its metabolites (means (±SD), medians (in cursive) and ranges (in parentheses)) detected in patients with the *SULT1A1*, *SULT1A2* and *SULT1E1* genotypes.

Genotype	Tamoxifen (ng/mL)	4-OH-tamoxifen (ng/mL)	N-desmethyl-tamoxifen (ng/mL)	Endoxifen (ng/mL)	Tamoxifen-N-oxide (ng/mL)
**SULT1A1**	200.92±86.14	8.44±7.30	458.09±209.96	22.43±17.79	48.69±18.93
**wt/wt**	*185.11*	*6.40*	*411.53*	*17.47*	*45.33*
n = 60	(88.08–576.23)	(1.06–30.96)	(35.96–1210.09)	(4.03–89.98)	(15.69–106.35)
**SULT1A1**	204.84±107.12	9.85±7.17	441.63±176.28	26.59±19.97	51.02±27.88
wt/*2	*171.44*	*7.98*	*418.46*	*22.58*	*41.84*
n = 46	(77.10–573.83)	(1.87–35.90)	(196.74–1191.13)	(4.18–107.10)	(17.43–142.97)
**SULT1A1**	205.87±101.52	9.20±6.80	459.42±160.94	28.68±24.18	56.02±24.50
*2/*2	*161.90*	*8.20*	*432.49*	*19.82*	*59.27*
n = 15	(102.43–396.73)	(1.47–29.54)	(212.67–782.92)	(4.57–95.87)	(15.69–99.37)
**SULT1A1 ** ***P value***	0.901	0.259	0.784	0.280	0.480
**SULT1A2**	189.75±71.12	6.02±3.28	428.02±162.17	17.21±11.79	47.65±19.19
**wt/wt**	*184.50*	*5.70*	*413.16*	*14.78*	*42.71*
n = 38	(77.10–375.04)	(1.47–14.95)	(35.96–847.98)	(4.03–58.77)	(15.69–99.37)
**SULT1A2**	227.33±131.55	11.38±9.34	478.01±247.44	28.84±23.78	60.87±31.96
wt/*2	*175.91*	*7.62*	*399.40*	*23.22*	*54.04*
n = 33	(88.08–576.23)	(2.12–35.90)	(212.67–1210.09)	(4.46–107.10)	(20.53–142.97)
**SULT1A2**	184.68±75.43	8.66±7.13	425.36±207.64	23.99±15.32	47.29±17.70
wt/*3	*174.42*	*7.20*	*324.21*	*19.02*	*46.20*
n = 16	(89.79–378.32)	(1.06–26.98)	(167.40–952.85)	(7.80–51.61)	(19.18–83.68)
**SULT1A2**	191.59±85.40	9.00±6.94	453.17±128.87	26.59±23.20	47.18±20.97
*2/*2	*173.20*	*7.52*	*427.03*	*20.76*	*50.56*
n = 16	(95.67–396.73)	(2.42–29.54)	(238.36–782.92)	(4.18–95.87)	(15.69–81.94)
**SULT1A2**	222.50±93.29	11.78±6.79	478.66±162.35	34.61±20.35	46.94±17.33
*2/*3	*240.78*	*11.20*	*527.52*	*32.02*	*48.81*
n = 13	(112.43–371.37)	(3.25–28.84)	(228.81–676.02)	(8.94–74.06)	(17.43–90.66)
**SULT1A2**	188.23±69.26	7.85±5.18	478.49±198.83	27.34±21.30	38.35±10.17
*3/*3	*164.35*	*6.54*	*485.02*	*21.02*	*34.87*
n = 5	(122.20–302.03)	(3.25–16.45)	(242.88–768.62)	(12.13–64.61)	(26.15–50.56)
**SULT1A2 ** ***P value***	0.905	0.056	0.815	0.027	0.546
**SULT1E1**	202.17±101.97	9.40±7.55	448.30±202.82	25.41±19.51	51.48±25.13
**wt/wt**	*172.65*	*7.26*	*399.40*	*19.12*	*45.33*
n = 88	(89.79–576.23)	(1.06–35.90)	(35.96–1.210.09)	(4.03–107.10)	(15.69–142.97)
**SULT1E1**	200.49±77.50	8.67±6.39	462.59±167.53	24.30±20.87	47.65±19.38
wt/*2	*186.14*	*6.20*	*426.41*	*18.04*	*47.94*
n = 28	(77.10–368.73)	(2.87–29.54)	(196.74–768.62)	(4.18–95.87)	(20.53–90.66)
**SULT1E1**	232.36±83.23	5.91±1.98	455.57±104.52	16.05±8.07	49.86±8.94
*2/*2	*202.74*	*5.70*	*497.51*	*20.60*	*45.33*
n = 5	(166.20–375.04)	(3.20–8.71)	(311.12–550.12)	(6.74–23.01)	(43.59–64.50)
**SULT1E1 ** ***P value***	0.424	0.614	0.720	0.557	0.835

**Figure 1:**
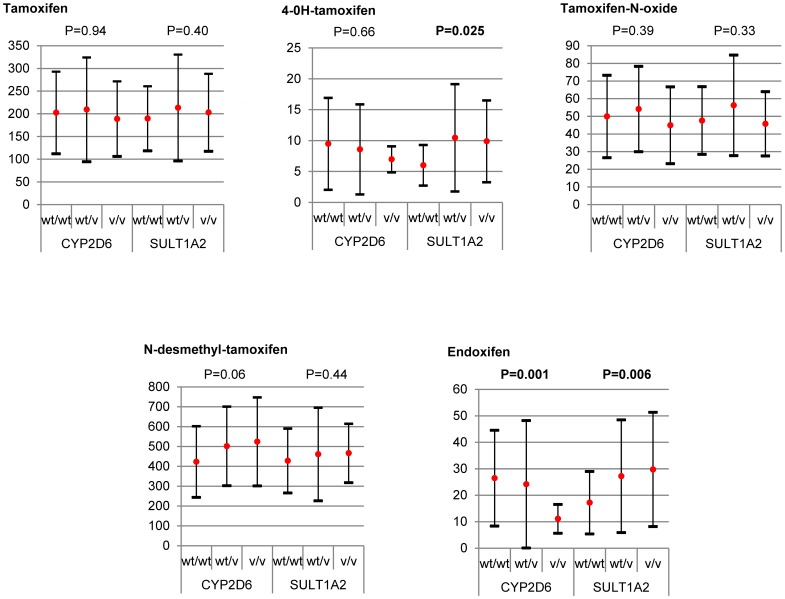
Concentrations of tamoxifen and its metabolites (means ± standard deviations, ng/mL) by *CYP2D6* and *SULT1A2* genotype subgroups established according to wt allele doses. *Sample sizes: CYP2D6:* wt/wt = 80; wt/v = 30; v/v = 11//*SULT1A2:* wt/wt = 38; wt/v = 49; v/v = 34.

As may be observed in Table 5, having optimal plasma concentrations of endoxifen seems dependent on carrying *CYP2D6* wt alleles and genotypes were accordingly classified into the three groups: wt/wt, patients with 2 or more copies of any functional allele; wt/v, patients carrying one functional allele and one variant -intermediate or null- allele; v/v, patients featuring intermediate or null alleles. Among these *CYP2D6* genotype subgroups, significant differences were only observed for the endoxifen metabolite (p = 0.001, Figure 1). Hence, when endoxifen concentrations were pairwise compared with these *CYP2D6* genotype groups, significantly lower values were detected in v/v than in wt/wt or wt/wt+wt/v (p<0.001 and p = 0.002, respectively). For the comparisons wt/wt *vs* wt/v and wt/v *vs* v/v, endoxifen concentrations were always lower in the groups with a smaller number of wt alleles though significance was not reached (p = 0.076 and p = 0.080, respectively). However, significant differences were observed when endoxifen/NDM-TAM ratios were compared among the same groups (p = 0.006 and p = 0.015, respectively). For the *SULT1A2* gene, patients were similarly stratified for comparisons, considering the significantly higher endoxifen levels observed in carriers of null alleles (Table 6). Thus, among the *SULT1A2* genotype subgroups (see Figure 1), significantly lower levels of 4OH-TAM and endoxifen were conferred by the wt/wt *SULT1A2* genotype (p = 0.025 and p = 0.006, respectively, Figure 1). Pairwise comparisons among these *SULT1A2* subgroups revealed significantly lower endoxifen levels in wt/wt compared to wt/v and v/v patients (p = 0.007 and p = 0.006, respectively). Further, similar results were obtained for the same pairwise comparisons for the 4OH-TAM metabolite (p = 0.022 and p = 0.012).

## Discussion

Prevalences of the commonly observed *CYP2D6* genotypes and alleles observed in our study are in good agreement with those reported for other European populations [29, for instance] with CYP2D6*4 being the most frequently detected null allele (12%, Table 4). Several studies examining worldwide genetic variation in the *CYP2D6* gene have revealed that this allele occurs most commonly in Caucasian populations (12%–21%) [30]. The prevalence of *CYP3A4* and *CYP3A5* variants in different populations varies considerably [31], [32]. The scarce prevalence of defective *CYP3A4* alleles in the subjects examined here is consistent with the data reported for other European populations of Caucasian origin [33]. In contrast, CYP3A5*3 polymorphism appears at a high incidence (97.78%, Table 4) in European populations, such as 94.35% in Greek [31], 94% in British [14] or 91.7% in Dutch [13] subjects. Similarly, *SULT* genes are polymorphic and show variable allele prevalences among ethnic groups [27]. The rates of the defective alleles SULT1A1*2, SULT1A2*3 and SULT1E1*2 determined in the present study are also in agreement with figures provided for other Caucasians populations [34,27,28, respectively].

The results of our study revealed plasma endoxifen concentrations of 24.75±19.37 ng/mL (mean) and 19.11 ng/mL (median) in 125 women under treatment with TAM, which is slightly lower than the levels detected by Borges et al. [35] and higher than those reported in other studies [36], [37]. Such variations are likely attributable to differences in sample handling, storage and measurement methods [38]. When endoxifen plasma concentrations were compared in patients taking or not taking selective serotonin reuptake inhibitors (CYP2D6 inhibitors), the difference was significant. Our patients who were taking both TAM and SSRIs were wt/wt except two who were wt/P for CYP2D6. These data are consistent with an effect of the *CYP2D6* genotype on endoxifen plasma concentrations, as described by other authors [39], [37], and suggest that pharmacogenetic variation in CYP2D6 activity may affect therapeutic outcomes of TAM treatment. However, larger trials are needed to determine the clinical implications of low circulating endoxifen concentrations.

When endoxifen plasma levels were compared according to the presence of two wt, one wt or no wt alleles, significant differences were detected in mean endoxifen concentrations between wt/wt *CYP2D6* and v/v *CYP2D6* patients (p<0.001). Other authors have also reported lower endoxifen concentrations in patients with the v/v *CYP2D6* genotype than those with the wt/wt genotype, regardless of the alleles tested [39], [40], [41], [42]. In our cohort, similar endoxifen levels were noted in patients showing the wt/wt or wt/v *CYP2D6* genotype, in accordance with the findings of other studies [41]. Although endoxifen concentrations differed between wt/v and v/v *CYP2D6* patients, significance was not reached (p = 0.080). Other authors have associated reduced CYP2D6 activity with a poor treatment outcome in terms of a higher risk of recurrence and shorter time of recurrence-free survival [43].

CYP3A4 and CYP3A5 contribute to the biotransformation of TAM into its primary metabolites: NDM-TAM and 4OH-TAM. We detected no differences in TAM metabolite levels for the variant alleles CYP3A4*1B, CYP3A4*3, CYP3A4*17 and CYP3A5*3 (Table 5). Although the possible relationship between CYP3A5*3 and TAM metabolism or clinical outcome of TAM therapy has been addressed, no significant link has been so far detected [44]. We should highlight the absence of the wt/wt *CYP3A5* genotype in our cohort of women with BC. This polymorphism should be assessed in future studies including a larger number of patients. According to the results obtained by Mugundu et al. [18], microsomes reveal a marked NDM-TAM reduction in wt/*3 and *3/*3 *CYP3A5* relative to wt/wt *CYP3A5*. In contrast, *CYP3A4* polymorphisms do not seem to be relevant in TAM metabolism, although some authors propose that certain combinations of *CYP3A4* and *CYP2C9* allelic variants may contribute to a TAM resistance phenotype [23].

While pharmacogenetic studies of SULTs have lagged behind studies examining other enzyme families, it is becoming increasingly clear that phase II drug metabolism plays an important role in the response shown by an individual to therapeutic agents. Sulfotransferases catalyze the formation of sulfated compounds of 4-hydroxytamoxifen and endoxifen [45]. The sulfation of a compound is considered to render it inactive, as sulfated molecules are poor ligands for the estrogen receptor [46]. However, prior studies have provided contradictory information. For instance, some authors observe no relation between *SULT1A1* genotypes and BC survival [22] while other authors have detected a strong link between survival and the common SULT1A1*1 allele, contrary to the expected outcome if a greater activity of the enzyme does in fact lead to rapid removal of the drug from target tissues [46]. In our study, no association between serum levels of TAM and its metabolites and SULT1A1 genotypes was observed (Table 6), as noted by others [47]. Despite some authors having described that some TAM metabolites, for instance 4OH-TAM, are more rapidly sulfated by SULT1E1 than by SULT1A1 [48], our results for the *SULT1E1* genotype were very similar to those recorded for *SULT1A1*, with TAM metabolite concentrations not varying significantly between wild type and null *SULT1E1* genotypes (Table 6).

SULT1A2 appears to be the most efficient human enzyme at sulfating several aromatic compounds [49]. Our results provide evidence that carriers of null *SULT1A2* alleles have significantly higher plasma levels of 4OH-TAM and endoxifen, the two hydroxylated substrates of the enzyme (p = 0.025 and p = 0.006, respectively, Figure 1). Although the findings of some studies have suggested a role of other SULT enzymes in TAM metabolism [22], [46], no previous study has addressed the relationship between SULT1A2 and plasma concentrations of TAM metabolites. Our results point to a possible benefit of carriers of alleles leading to lower enzyme activity levels (SULT1A2*2 and SULT1A2*3) in maintaining optimal levels of 4OH-TAM and endoxifen. Thus, significant higher 4OH-TAM levels were recorded here in wt/v and v/v than in wt/wt *SULT1A2* patients (p = 0.007 and p = 0.006, respectively). Similar results were obtained for endoxifen levels (p = 0.022 and p = 0.012, respectively). Consequently, only one defective *SULT1A2* allele seems to be sufficient to slow down the conversion of the two hydroxylated substrates into sulfonated substrates. When *CYP2D6* and *SULT1A2* were considered together, it was observed that some allelic variant combinations of both genes seem to markedly affect an individual’s response to TAM therapy, as noted by other authors [23]. Accordingly, the wt/wt *CYP2D6* genotype gave rise to endoxifen levels of over two-fold those observed in patients with the v/v *CYP2D6* genotype and the wt/v and v/v *SULT1A2* genotypes rendered significantly higher levels of both endoxifen and 4OH-TAM (Figure 1). One of the limitations of our study was the sample size, although the number of patients analyzed is similar to those included in other studies [43], [45], [47]. However, the small numbers of some classes of genotypes compared might have led to a low statistical power in some of the tests. It could therefore be that some differences would have emerged if we had data for a larger patient cohort. This issue will be no doubt resolved in future studies.

In conclusion, our findings indicate that besides the *CYP2D6* genotype inducing the conversion of TAM to potent hydroxylated metabolites in a manner consistent with a gene-dose effect, the *SULT1A2* genotype also seems to play an important role in maintaining optimal levels of both 4OH-TAM and endoxifen. Consequently, patients who are wt/wt for *CYP2D6* and also feature the SULT1A2*2 or SULT1A2*3 alleles could be the best candidates for a good response to TAM therapy in terms of eliciting adequate plasma endoxifen and 4OH-TAM levels. Indeed, *CYP2D6* and *SULT1A2* genotype distributions may partly explain the wide interindividual variations detected in the pharmacokinetics of TAM. Given that several other drugs and enzymes may also affect TAM metabolism, we recommend therapeutic drug monitoring in TAM trials designed to assess the effects of both *CYP2D6* and *SULT1A2* genotypes on treatment outcome.
